# TOP1 CAD-seq: A protocol to map catalytically engaged topoisomerase 1 in human cells

**DOI:** 10.1016/j.xpro.2022.101581

**Published:** 2022-08-01

**Authors:** Vladislav Kuzin, Anika Wiegard, Donald P. Cameron, Laura Baranello

**Affiliations:** 1 Department of Cell and Molecular Biology, Karolinska Institutet, 17177 Stockholm, Sweden

**Keywords:** Bioinformatics, Cancer, Sequencing, ChIPseq, Molecular Biology, Antibody

## Abstract

TOP1 CAD-seq enables mapping of TOP1 sites of covalent engagement with DNA. The procedure depends upon enrichment of DNA-covalent adducts using chaotropic salts and immunoprecipitation with an antibody specific for TOP1. Here, we describe a step-by-step protocol compatible with Illumina sequencing and bioinformatic pipeline for preliminary data analysis. Compared to other approaches for the genomic study of topoisomerases, TOP1 CAD-seq provides information about active TOP1 engaged on the DNA, taking advantage of low background due to absence of crosslinking.

For complete details on the use and execution of this protocol, please refer to Das et al. (2022).

## Highlights

• TOP1 CAD-seq maps sites of topoisomerase 1 catalytic engagement with the DNA
• The absence of a cross-linking step reduces the background signal
• The method is high throughput and takes no longer than a regular ChIP-seq protocol
• The method can be used as a proxy to assess the level of supercoiling in the genome

## Abstract (editor highlights)

Publisher’s note: Undertaking any experimental protocol requires adherence to local institutional guidelines for laboratory safety and ethics.

## Graphical abstract

**Figure:**
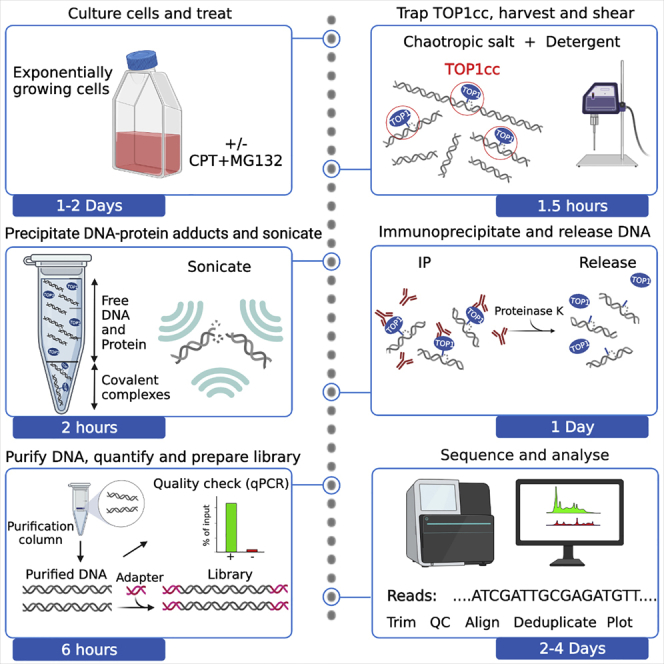

## Before you begin

### Introduction

It has become increasingly evident that DNA topology plays an active role in regulating nuclear processes such as transcription, replication, chromatin remodeling and genome folding (Baranello et al., 2016; Guo et al., 2021; Heintzman et al., 2019; Herrero-Ruiz et al., 2021; Kouzine et al., 2013b, 2014; Rani and Nagaraja, 2019; Salceda et al., 2006; Teves and Henikoff, 2014). Nevertheless, the complex interplay between DNA topology and DNA processes, combined with technological barriers to assess DNA topological changes in the genome, have so far prevented rapid progress in this area.

Topoisomerases (TOP1 and TOP2) are fundamental enzymes that control DNA topology. They remove the torsional stress in the genome by a mechanism involving cleavage and resealing of one (TOP1) or two (TOP2) DNA strands (Pommier et al., 2016; Wang, 1996). During the cleavage-religation cycle, topoisomerases form a covalent link with the DNA phosphodiester backbone generating the topoisomerase cleavage complex (TOPcc). This complex is extremely transient in cells, unless stabilized with topoisomerase poisons and inhibitors of proteasomal degradation (Sciascia et al., 2020). The detection of TOPccs on the DNA could be used as a proxy to measure the torsional stress in the genome.

Several techniques are available to measure TOPccs. The Rapid Approach to DNA Adduct Recovery (RADAR) (Fielden et al., 2020; Kiianitsa and Maizels, 2013) and similar techniques (Anand et al., 2018; Herrero-Ruiz et al., 2021) enable immunodetection of topoisomerase-DNA covalent adducts in population of cells treated with topoisomerase poisons. Although commonly used, these approaches do not inform about topoisomerase activity on a genomic scale. Other methods exist to indirectly map TOPccs. For example CC-seq (Gittens et al., 2019) can be used to detect all covalent DNA-protein complexes genome wide, while BLISS (Yan et al., 2017) and END-seq (Canela et al., 2016; Wong et al., 2021) allow for mapping of TOP2-related DNA double strand breaks. Because these methods do not include an antibody immunoprecipitation step, they inform about the whole population of protein-DNA adducts or DNA double strand breaks in cells, without discriminating between the activities of single topoisomerases or other proteins. In contrast, topoisomerase ChIP-seq, which is based on immunoprecipitation with an anti-topoisomerase specific antibody, has improved specificity. However, the inclusion of a crosslinking step results in higher background because any topoisomerase in the vicinity of DNA will be detected. Techniques such as CUT&RUN (Skene and Henikoff, 2017) or CUT&TAG (Kaya-Okur et al., 2019) can improve the signal to noise ratio and present a more physiologically relevant picture by showing the native binding state of topoisomerase on the DNA in the absence of crosslinking. However, these approaches inform about the total population of topoisomerase bound to chromatin, regardless of whether it is actively engaged with the DNA or not. Our previously developed TOP1-seq (Baranello et al., 2016), a native TOP1 ChIP-seq approach, is suitable to detect TOP1 catalytically engaged with the DNA, but involves numerous steps during library preparation, including two sequential ligations, that affect the overall time, background noise and yield.

We instead propose a simple solution to the problem. By combining the RADAR approach with antibody immunoprecipitation and sequencing we developed Covalent Adduct Detection of topoisomerase and sequencing (TOP CAD-seq), to measure TOPccs genome wide. Harvesting cellular lysates in the presence of chaotropic salts and detergents, stabilizes protein-DNA adducts, including TOPccs (Kiianitsa and Maizels, 2013). Performing precipitation of protein-DNA adducts before addition of the specific antibody, significantly concentrates the amount of TOPccs for further immunoprecipitation, and therefore increase the DNA recovery relative to input. Lastly, we use a simplified protocol for library preparation to improve yield. We have tested the protocol for detection of TOP1ccs both in suspension (K562) and adherent (HCT116) cells with high reproducibility. The results reported here correlate with our published TOP1-seq data (Baranello et al., 2016) showing a drop of TOP1 activity at transcription start sites (TSSs) and increased activity downstream of TSSs. Since TOP1 CAD-seq provides a measurement of TOP1 activity across the genome, it can be used as a surrogate for assessment of DNA torsional stress. TOP1 CAD-seq gives higher signal to noise ratio and is faster than TOP1-seq. From cell harvest to library preparation, it takes only 2 days. Of note, the method can be applied to various model systems where the RADAR assay has been validated (e.g., bacteria or yeast (Aldred et al., 2019; Anand et al., 2018)) and can be implemented for the study of other topoisomerases, provided that ChIP-grade antibodies are available.

### Preparation step: Cell culture

**Timing: 1–2 Days**

Seed the cells to reach 60% confluency on the day of harvest. We suggest using at least 1 × 10^7^ cells for each immunoprecipitation (IP) and to perform all treatments at least in duplicates.

***Note:*** Consider preparing a higher number of cells if you want to include an additional negative control such as IP with IgG antibody (sc-2025).

1. Set the incubator to 37°C, 5% CO_2_, at least 95% humidity. Culture K562 cells in RPMI (Thermo Fisher Scientific, 21875034) media containing FBS 10% (Thermo Fisher Scientific, 26170043), 2 mM GlutaMAX™ (Thermo Fisher Scientific, 35050061) and 1 mM sodium pyruvate (Sigma, S8636).

***Optional:*** Add Penicillin-Streptomycin (Thermo Fisher Scientific, 15140122) for an additional layer of protection from bacterial contamination.

***Note:*** Cell types expressing low levels of TOP1 are not suitable for the assay. We advise to assess TOP1 protein levels by Western blot before starting the protocol. TOP1 levels from HCT116 or K562 cells can be used as a reference.

2. Seed 1 × 10^7^ exponentially growing cells in T175 flasks (20 mL media with ∼0.5 × 10^6^ cell/mL).
3. 24 h after seeding, count the cells and prepare to harvest if they have reached ∼1 × 10^6^ cell/mL.

***Note:*** Cellular viability is important for the success of TOP1 CAD-seq. Start with high-quality healthy cells characterized by at least 90% viability.

***Note:*** We advise to use a low passage cell culture to prevent passage-related effects.

***Note:*** It is also important to harvest cells during the exponential phase to ensure that cells maintain high levels of transcription and replication and have proper expression of TOP1. Harvesting under- or over-confluent cells might bias the results.

Alternative for adherent cells:

***Note:*** Choose appropriate culture conditions and media depending on the cell line.

4. Seed the cells to have them 60% confluent on the day of harvest.

***Note:*** Prepare one extra flask, which will be used for cell counting on the day of harvest.

***Note:*** For adherent cells, confluency can be approximated by looking cells at the microscope and assessing the relative area coverage.

5. 24 h after seeding, check the confluency by trypsinizing cells in one of the flasks and counting. Continue if you have at least 1 × 10^7^ cells for each sample.

## Key resources table

**Table:** 

REAGENT or RESOURCE	SOURCE	IDENTIFIER
**Antibodies**		

Rabbit monoclonal Anti-Topoisomerase I antibody [EPR5375]	Abcam	Cat# ab109374; RRID: AB_10861978
normal mouse IgG	Santa Cruz	Cat# sc-2025; RRID: AB_737182
Goat Anti-Rabbit IgG H&L (HRP) antibody	Abcam	Cat# ab205718, RRID: AB_2819160

**Chemicals, peptides, and recombinant proteins**		

RPMI 1640 Medium	Thermo Fisher Scientific	Cat# 21875034
Bovine Serum, heat inactivated, New Zealand origin	Thermo Fisher Scientific	Cat# 26170043
GlutaMAX™ Supplement	Thermo Fisher Scientific	Cat# 35050061
Sodium pyruvate solution	MiliporeSigma	Cat# S8636
Penicillin-Streptomycin (10,000 U/mL)	Gibco	Cat# 15140122
cOmplete™, Mini, EDTA-free Protease Inhibitor Cocktail	MiliporeSigma	Cat# 04693132001
4-(2-Aminoethyl)benzenesulfonyl fluoride hydrochloride (AEBSF)	MiliporeSigma	Cat# A8456
MG132(R)	Sigma	Cat# SML1135
Pierce™ Protein A/G Magnetic Beads	Thermo Fisher Scientific	Cat# 88803
UltraPure™ Agarose	Thermo Fisher Scientific	Cat# 16500500
Camptothecin	MiliporeSigma	Cat# C9911-250MG
Ethidium bromide solution	MiliporeSigma	Cat# E1510
Proteinase K, Molecular Biology Grade	New England Biolabs Inc.	Cat# P8107S
Phenol:Chloroform:Isoamyl Alcohol 25:24:1 Saturated with 10 mM Tris, pH 8.0, 1 mM EDTA	MiliporeSigma	Cat# P2069-400ML
Sodium dodecyl sulfate	MiliporeSigma	Cat# L3771-100G
Lithium chloride anhydrous, free-flowing, Redi-Dri™, ReagentPlus®, 99%	MiliporeSigma	Cat# 793620-100G
Glycerol for molecular biology, ≥99.0%	MiliporeSigma	Cat# G5516-1L
Triton™ X-100 for molecular biology	MiliporeSigma	Cat# T8787-50ML
*N*-Lauroylsarcosine sodium salt (Sarkosyl) BioXtra, ≥97% (TLC)	MiliporeSigma	Cat# L5777-50G
Sodium deoxycholate monohydrate BioXtra, ≥99.0% (titration)	MiliporeSigma	Cat# D5670-25G
DTT 1,4-Dithiothreitol	MiliporeSigma	Cat# 11583786001
Nonidet™ P 40 Substitute	MiliporeSigma	Cat# 74385
Ethylenediaminetetraacetic acid disodium salt dihydrate	MiliporeSigma	Cat# 03685-500G
Fast SYBR™ Green Master Mix	Thermo Fisher Scientific	Cat# 4385618
Exonuclease VII	New England Biolabs Inc.	Cat# M0379S
AMPure XP beads	Beckman	Cat# A63880
SYBR™ Green I Nucleic Acid Gel Stain - 10,000× concentrate in DMSO	Thermo Fisher Scientific	Cat# S7563
GeneRuler 1 kb DNA Ladder	Thermo Fisher Scientific	Cat# SM0311
Ethanol absolute ≥99.8%, Electran Molecular biology grade	VWR	Cat# 437435L
RNase, DNase-free	Roche	Cat# 11119915001

**Critical commercial assays**		

Agilent High Sensitivity DNA Kit	Agilent	Cat# 5067-4626
NextSeq 500/550 High Output Kit v2.5 (75 Cycles)	Illumina	Cat# 20024906
MiniElute PCR Purification Kit	QIAGEN	Cat# 28006
ThruPLEX DNA-seq kit	Takara	Cat# R400676
Qubit™ 1× dsDNA HS Assay Kit	Thermo Fisher Scientific	Cat# Q33230

**Deposited data**		

Raw and processed sequencing data	Das et al. (2022)	GEO: GSE181448
RNA-seq data	Goldenson et al. (2020)	GEO: GSE150806

**Experimental models: Cell lines**		

HCT116	Developmental Therapeutics Programme. Details: Human cells isolated from the colon of an adult male, colon cancer patient. The cells have a mutation in codon 13 of the Ras proto-oncogene (karyotype 45).	N/A
K562 MYCmAIDTir1-eBFP2 (K562MYC_mAID)	Gift from Dr. J. Zuber (IMP, Austria). Details: K562 cells are established from pleural effusion of 53-year-old female with chronic myelogenous leukemia in terminal blast crisis (karyotype 2n = 46).	N/A

**Oligonucleotides**		

qPCR forward primer for MYC	GGACTCAGTCTGGGTGGAAGG	N/A
qPCR reverse primer for MYC	AAGGAGGAAAACGATGCCTAGA	N/A

**Software and algorithms**		

GraphPad Prism 9.1.0 (software for graph and statistics analysis	GraphPad	https://www.graphpad.com/scientific-software/prism/; RRID: SCR_002798
BioRender	BioRender	https://biorender.com/; RRID: SCR_018361
bcl2fastq v2.20.0.422	Illumina	https://emea.support.illumina.com/sequencing/sequencing_software/bcl2fastq-conversion-software.html; RRID: SCR_015058
Bowtie2 2.3.5.1	Langmead and Salzberg (2012)	http://bowtie-bio.sourceforge.net/bowtie2/index.shtml; RRID: SCR_016368
Cutadapt 3.1	Martin, 2011	https://cutadapt.readthedocs.io/en/stable/; RRID: SCR_011841
Deeptools 2.5.0	Ramírez et al., 2014	https://deeptools.readthedocs.io/en/develop/; RRID: SCR_016366
FastQC 0.11.9	https://www.bioinformatics.babraham.ac.uk/projects/fastqc/	https://github.com/s-andrews/FastQC; RRID: SCR_014583
MultiQC 1.10	Ewels et al. (2016)	http://multiqc.info/; RRID: SCR_014982
Picard tools 2.10.3	Broad Institute	https://broadinstitute.github.io/picard/; RRID: SCR_006525
Samtools 1.8	Li et al., 2009	http://www.htslib.org/; RRID: SCR_002105
ngsplot 2.61	Shen et al. (2014)	https://github.com/shenlab-sinai/ngsplot; RRID: SCR_011795
EnrichedHeatmap 1.22.0	Gu et al. (2018)	https://bioconductor.org/packages/release/bioc/html/EnrichedHeatmap.html

**Other**		

milliTUBE 1 mL AFA Fiber	Covaris	Cat# 520130
ME220 Focused-ultrasonicator	Covaris	Cat# 500506
NextSeq 550 Sequencing System	Illumina	Cat# SY-415-1002
2100 Bioanalyzer Instrument	Agilent	Cat# G2939BA
Bioruptor® Sonication System	Diagenode	Cat# UCD-200
Sonopuls HD 2070.2 Ultrasonic homogenizer	BANDELIN	Cat# 2451
MS 73 ultrasonic probe	BANDELIN	Cat# 529
Qubit™ 3 Fluorometer	Invitrogen	Cat# Q33216

## Materials and equipment

**Table:** AEBSF 416×

Reagent	Final concentration	Amount
AEBSF (239.69 g/mol)	208.6 mM (416×)	50 mg
Deionized water		1 mL
**Total:**		1 mL

***Note:*** Final concentration of AEBSF in all solutions where it is listed is 1× (0.5 mM).

Store at −20°C for one year.

**Table:** PI (proteinase inhibitor cocktail) 50×

Reagent	Final concentration	Amount
EDTA-free Protease Inhibitor Cocktail	50×	1 tablet
Deionized water		1 mL
**Total:**		1 mL

***Note:*** Final concentration of PI in all solutions where it is listed is 1×.

Store at −20°C for one year.

**Table:** Buffer M (incomplete)

Reagent	Final concentration	Amount
Tris-HCl pH 6.5 (1 M)	9.3 mM	0.5 mL
EDTA (0.5 M)	18.6 mM	2 mL
Guanidine thiocyanate (GTC, powder)	5.59 M	35.46 g
Deionized water		Fill up to 50 mL
**Subtotal**	**N/A**	**50 mL**

***Note:*** Prior to addition of DTT/Sarcosyl/Triton, you can store the buffer at 25°C (this is our room temperature) for six months.

***Note:*** Because Buffer M can precipitate, prior to addition of DTT/Sarcosyl/Triton, place the buffer at 37°C to dissolve the crystals, at least 1 h before starting the experiment. Immediately before harvesting, add DTT/Sarcosyl/Triton and finally supplement with proteinase inhibitor cocktail (1× final concentration).

**Table:** Buffer M (complete)

DTT (powder)	0.93%	0.5 g
Sarcosyl (30%)	0.93%	1.66 mL
Triton X-100	3.72%	1.5 mL
Proteinase inhibitor cocktail 50×	1× (optionally put one tablet in 50 mL solution without preparing 50× stock)	1 mL
**Total**	**N/A**	**54.16 mL**

Discard the excess of final buffer after use, do not store.

**CRITICAL:** Perform weighting of GTC and DTT powders on table with downdraft ventilation or in fume hoods to avoid inhalation of powder particles.

**Table:** Wash buffer

Reagent	Final concentration	Amount
Tris-HCl pH 7.5 (2 M)	20 mM	1 mL
EDTA (0.5 M)	1 mM	0.2 mL
NaCl (4 M)	50 mM	1.25 mL
Ethanol 100%	50%	50 mL
Deionized water		47.55 mL
**Total**	**N/A**	**100 mL**

Store at 4°C for six months.

**Table:** RIPA

Reagent	Final concentration	Amount
Tris-HCl pH 8.0 (1 M)	10 mM	0.5 mL
EDTA (0.5 M)	1 mM	0.1 mL
Triton X-100	1%	0.5 mL
SDS 10%	0.1%	0.5 mL
NaCl (4 M)	200 mM	2.5 mL
Na-Deoxycholate 5%	0.1%	1 mL
Deionized water		44.9 mL
**Total**	**N/A**	**50 mL**

Store at 4°C for six months.

**Table:** RIPA 5×

Reagent	Final concentration	Amount
Tris-HCl pH 8.0 (1 M)	10 mM	0.5 mL
EDTA (0.5 M)	1 mM	0.1 mL
Triton X-100	5%	2.5 mL
SDS 10%	0.1%	0.5 mL
NaCl (4 M)	1 M	12.5 mL
Na-Deoxycholate 5%	0.5%	5 mL
Deionized water		28.9 mL
**Total**	**N/A**	**50 mL**

Store at 4°C for six months.

**Table:** RIPA300

Reagent	Final concentration	Amount
Tris-HCl pH 8.0 (1 M)	10 mM	0.5 mL
EDTA (0.5 M)	1 mM	0.1 mL
Triton X-100	1%	0.5 mL
SDS 10%	0.1%	0.5 mL
NaCl (4 M)	300 mM	3.75 mL
Na-Deoxycholate 5%	0.1%	1 mL
Deionized water		43.65 mL
**Total**	**N/A**	**50 mL**

Store at 4°C for six months.

**Table:** LiCl-SDS 0.1%

Reagent	Final concentration	Amount
Tris-HCl pH 8.0 (1 M)	10 mM	0.5 mL
EDTA (0.5 M)	1 mM	0.1 mL
LiCl (8 M)	250 mM	1.5625 mL
NP40	0.5%	0.25 mL
Na-Deoxycholate 5%	0.5%	5 mL
SDS 10%	0.1%	0.5 mL
Deionized water		42.0875 mL
**Total**	**N/A**	**50 mL**

Store at 4°C for six months.

**Table:** TE

Reagent	Final concentration	Amount
Tris-HCl pH 8.0 (1 M)	10 mM	0.5 mL
EDTA (0.5 M)	1 mM	0.1 mL
Deionized water		49.4 mL
**Total**	**N/A**	**50 mL**

Store at 4°C for six months.

**Table:** TE-SDS 0.1%

Reagent	Final concentration	Amount
Tris-HCl pH 8.0 (1 M)	10 mM	0.5 mL
EDTA (0.5 M)	1 mM	0.1 mL
SDS 10%	0.1%	0.5 mL
Deionized water		48.9 mL
**Total**	**N/A**	**50 mL**

Store at 4°C for six months.

**Table:** TE-NaCl

Reagent	Final concentration	Amount
Tris-HCl pH 8.0 (1 M)	10 mM	0.5 mL
EDTA (0.5 M)	1 mM	0.1 mL
NaCl (4 M)	0.5 M	6.25 mL
Deionized water		43.15 mL
**Total**	**N/A**	**50 mL**

Store at 25°C for one year.

**Table:** DNA gel loading buffer 10×

Reagent	Final concentration	Amount
Sucrose/Glycerol	60%	600 μL
Bromophenol blue (10%)	0.01%	10 μL
Tris-HCl pH7.5 (1 M)	10 mM	10 μL
Deionized water		380 μL
**Total**	**N/A**	**1 mL**

Store at 25°C for one year.

**Table:** TBS

Reagent	Final concentration	Amount
Tris-HCl pH7.5 (1 M)	100 mM	5 mL
NaCl (4 M)	150 mM	1.875 mL
Deionized water		43.125 mL
**Total**	**N/A**	**50 mL**

Store at 25°C for one year.

## Step-by-step method details

### Cell treatment and harvest

**Timing: 1 h**

Because the TOP1ccs are short-lived, treat cells with the proteasome inhibitor MG132 and TOP1 poison camptothecin (CPT) to trap the TOP1ccs and prevent their proteolytic degradation (Sciascia et al., 2020). After treatment, harvest the cells in Buffer M containing a high concentration of chaotropic salts (GTC) to stabilize the protein-DNA adducts (including the TOP1 molecules engaged with the DNA) and to remove all non-covalently bound proteins.

**CRITICAL:** Due to the transient nature of TOP1ccs, small differences in handling samples during this step might result in high variability. It is very important to be consistent with the time of treatment and harvest.

**CRITICAL:** While it is recommended to work at cold temperature (10°C), it is also important to not place the sample in direct contact with ice since Buffer M easily precipitates (troubleshooting 1).

***Note:*** As this is the most crucial and time-sensitive step, make sure you have the following reagents prepared and thawed before starting: MG132, CPT, Buffer M (without precipitates). Consider the time needed for transporting the cells from/to the incubator. Stagger the samples to improve consistency.

***Note:*** Replace MG132 and CPT with an equivalent volume of DMSO for negative control sample.

1. After counting the K562 cells, transfer the appropriate amount (10 mL of ∼1 × 10^6^ cell/mL per IP) in a 50 mL falcon tube and perform all the treatments/incubations (steps 2–4) in that tube.
2. Treat the cells with 10 μM MG132 (Sigma, SML1135) or with an equivalent volume of DMSO for the negative control sample.
  a. Add 4 μL of MG132 (50 mM stock) [or DMSO] to the media (20 mL of ∼1 × 10^6^ cell/mL, 2 × 10^7^ cells in total) and resuspend by inverting the tube.
  b. Incubate at 37°C, 5% CO_2_ for 30 min.
3. Treat with 20 μM CPT (Sigma, C9911) [or DMSO for the negative control].
  a. Add 40 μL of CPT (10 mM stock) [or DMSO] directly to the media and resuspend by inverting the tube.
  b. Incubate at 37°C for 5 min.
4. Pellet the cells and add Buffer M + protein inhibitors cocktail (1× final concentration, Sigma, 04693132001) to the tube.
  a. Spin down the cells at 300 g for 3 min.
  b. Remove the supernatant, by pouring out the media and (while the tube is still upside down) quickly blot the excess of the media on a paper tissue. Be careful not to disturb the cell pellet.
  c. Immediately add 5 mL of Buffer M + protein inhibitors cocktail.
5. Carefully mix the sample with a 5 mL serological pipette and split into two 15 mL falcon tubes, aliquoting ∼2.5 mL sample in each. Place the tubes in a rack on top of ice.

**CRITICAL:** Do not let the tubes directly touch the ice to avoid precipitation of Buffer M.

**CRITICAL:** Although very viscous, try to evenly distribute the suspension in the tubes.

Alternative if you are working with adherent cells:

6. Treat the cells with 10 μM MG132 or with an equivalent volume of DMSO for the negative control sample (Methods video S1):
  a. Transfer the media from the flask into a 50 mL falcon tube.
  b. Add 4 μL of MG132 (50 mM stock) [or DMSO] to the media (20 mL) and resuspend by inverting the tube.
  c. Carefully add the media back into the flask.
  d. Incubate at 37°C for 30 min.
7. Treat with 20 μM CPT [or DMSO for the negative control] (Methods video S2):
  a. Tilt the flask on the side to collect the media in a corner close to the lid and directly add 40 μL of CPT (10 mM stock) [or DMSO] to that media.
  b. Move the flask gently to evenly distribute CPT within media.
  c. Incubate at 37°C for 5 min.

Methods video S1. MG132 treatment of adherent cells, related to Cell treatment and harvest: step 6

8. Remove all the media from the flask and add Buffer M + protein inhibitors cocktail as quick as possible (Methods video S3):
  a. Pour out the media from the flask.
  b. Tilt the flask to collect the leftover media in one corner and remove it with a serological pipette.
  c. Add 5 mL Buffer M + protein inhibitors cocktail and make sure that it distributes evenly in the flask.
  d. Scrape the cells and collect the suspension into 15 mL falcon tubes. Place the tubes in a rack on top of ice.

Methods video S2. CPT treatment of adherent cells, related to Cell treatment and harvest: step 7

9. Carefully mix the sample with a 5 mL serological pipette and split into two 15 mL falcon tubes, aliquoting ∼2.5 mL sample in each. Place the tubes in a rack on top of ice.

**CRITICAL:** Do not let the tubes directly touch the ice to avoid precipitation. Do not place the cell culture flasks with Buffer M + protein inhibitors cocktail on ice for longer than 30 s, because the buffer will precipitate.

**CRITICAL:** Although very viscous, try to evenly distribute the suspension in the tubes.

Methods video S3. Removal of media and addition of buffer M to adherent cells, related to Cell treatment and harvest: step 8

### Pre-sonication with a probe

**Timing: 5 min × (number of samples)**

To solubilize the samples and facilitate the following precipitation, fragment the DNA. Several short pulses with a Sonopuls HD 2070.2 probe sonicator (Bandelin, 2451) with MS-73 probe (Bandelin, 529) significantly increase the precipitation efficiency.

***Alternatives:*** You can use other probe sonicators, upon previous optimization.

**CRITICAL:** Avoid heating the sample. Do not sonicate for more than 30 s at a time. Include 30 s pause in between sonication cycles for the temperature to cool down. Add more ice to the water if it melts completely.

10. Sonicate the samples at 20% amplitude in a 1 s ON 1 s OFF mode for 3 × 30 s with a 30 s pause in between each sonication cycle.

**CRITICAL:** During the sonication, put the falcon tube in a rack in cold water and add ice around the rack. Avoid having direct contact of ice with the tube, as Buffer M can easily precipitate. If this happens, follow troubleshooting Problem 1: Precipitation.

**CRITICAL:** Place the tip of the probe about 0.5 cm from the bottom of the tube and avoid foaming of the sample. If foam forms, follow troubleshooting Problem 2: Foaming.

***Note:*** Between the sonication of the different samples, rinse the probe sonicator first with SDS 0.1% in ultrapure water and then with ultrapure water.

***Note:*** With probe sonicators, 2.5 mL in a 15 mL Falcon allows for optimal sonication. Do not increase the volume of the sample above this threshold. Divide samples with bigger volumes in 2.5 mL aliquots.

***Note:*** Over time probe tips degrade and the sonication efficiency decreases. If this happens, increase the amplitude of sonication or replace the probe.

11. After sonicating pulse-spin the tubes in a pre-cooled centrifuge to collect all material and pool both 2.5 mL aliquots in the same tube.

### Precipitation

**Timing: 1 h**

Add 0.5 volume of 100% cold Ethanol to precipitate the DNA-covalent adducts.

**Pause point:** Optionally after precipitation and resuspension in TE-SDS 0.1% you can freeze the samples at −80°C and continue later.

***Note:*** Always keep the samples on ice or in cooled racks.

***Note:*** If you freeze the sample, do not store it for longer than 5 days.

12. Precipitate all the samples with 0.5 volumes of 100% cold Ethanol (VWR, 437435L).
  a. Aliquot 1.3 mL of the samples in 2 mL Eppendorf tubes.
  b. Add 650 μL of 100% cold Ethanol to each tube.
  c. Mix and incubate at −20°C for at least 10 min.
  d. Spin the samples at 21,000 g (or maximum speed), 4°C for 10 min.
13. Remove the supernatant and wash the pellet three times with 800 μL of cold Wash Buffer.
  a. Remove the supernatant by pouring it out.
  b. Add 800 μL of cold Wash Buffer and vortex the pellet shortly (until it detatches) at high setting to facilitate mixing with the Wash Buffer.
  c. Spin down the sample at 21,000 g (or maximum speed) and 4°C for 10 min.
  d. Repeat steps 13a–13c two more times.
14. Pour out the supernatant and remove as much Wash Buffer as possible. Specifically, after the final wash, decant the supernatant, keep the tube inverted and drain the excess of liquid on a tissue. Finally, delicately aspirate the remaining liquid with a vacuum device (Methods video S4):

**CRITICAL:** Be careful not to touch the pellet during aspiration with the vacuum device. Avoid shaking or inverting the tube after decanting.

15. Dry the pellet for 5 min at 25°C.

***Note:*** Be careful to not over-dry the pellet. Longer drying might impair the resuspension of the pellet.

***Note:*** When dry, the pellet becomes transparent.

16. Resuspend each pellet in 500 μL of TE-SDS 0.1% + protein inhibitors cocktail + AEBSF (0.5 mM, Sigma, A8456).
17. Leave the aliquots on ice for 5 min (the pellet will start to dissolve) and then mix with a pipette to resuspend the pellet. Pool the aliquots from the same samples together and rotate the sample at 4°C for 30 min or until they become completely resuspended.

***Note:*** In case of problems with resuspension follow troubleshooting Problem 3: Resuspension.

Methods video S4. Using vacuum source to remove residual Ethanol from the pellet, related to Precipitation: step 14

### Sonication with Covaris

**Timing: 10 min × (number of samples)**

For efficient IP and further library preparation sonicate to achieve an optimal fragment size below 1000 bp. Sonication with Covaris ME220 (Covaris, 500506) has been proven very effective for fragmentation of samples prepared for sequencing.

***Alternatives:*** Other sonicators with similar properties might work upon optimization.

***Note:*** While you wait for the samples to sonicate, we advise to start preparing the antibody-beads complexes. In that case, after you start the sonication, follow Immunoprecipitation with anti-TOP1 antibody, part I:

***Note:*** Always keep the samples on ice or in cooled racks.

**Pause point:** Optionally, after sonication you can freeze the samples at −80°C and continue with the IP on the next day or within up to three days. However, if you decide to freeze the sample, we suggest doing it before sonication with Covaris, as the TOP1ccs are more stable.

18. Sonicate each sample for 5 min with Covaris.
  a. Transfer 1 mL samples into Covaris milliTUBE 1 mL with AFA Fiber (Covaris, 520130).
    ***Note:*** Fill up the tube to the very top and remove bubbles. If necessary, add more TE-SDS 0.1% + protein inhibitors cocktail + AEBSF before carefully screwing the cap.
    **CRITICAL:** It is important to not have any bubbles inside the tube because they can decrease the efficiency of sonication. Check for absence of bubbles by gently inverting the tube.
  b. Set up the “High Cell” protocol for Covaris ME220 and sonicate the samples for 5 minutes.

**Table:** High Cell protocol

Settings	Value
Duration, sec	300
Peak Power	75.0
Duty Factor, %	15
Cycles/Burst	1000
Avg Power	11.25
Temp Setpoint, C	9.0
Temp Range, C	4.0–14.0

19. After sonication, transfer 20 μL of each sample into 1.5 mL Eppendorf tube. Use this material to assess the size of the DNA fragments on an agarose gel, follow Assessing fragmentation on an agarose gel:. Keep the remaining sample on ice or at 4°C in the fridge.

### Immunoprecipitation with anti-TOP1 antibody, part I

**Timing: 4 h**

***Optional:*** Perform this step during the sonication, so that you will have the antibody-beads complexes ready to start the IP once the samples are sonicated and the fragment size is verified.

***Note:*** Always keep the samples on ice or in cooled racks.

20. Transfer 25 μL × (number of samples + 10%) of Protein A/G beads (Thermo Fisher Scientific, 88803) to a 1.5 mL Eppendorf tube.

***Note:*** Do not transfer more than 150 μL of beads (6 samples) in one 1.5 mL tube because the beads will be later diluted in 200 μL × (number of samples + 10%).

***Note:*** Mix the stock of beads by inverting at least 20 times and vortexing gently (until visually homogenous) before pipetting.

***Note:*** Perform all pipetting of Protein A/G beads using filter tips with the tip cut off using sterile/clean scissors to prevent clogging of the tip and avoid shearing forces on the beads.

***Note:*** Preparing 10% extra beads will also limit the possibility of running out of beads in the last aliquot due to pipetting variability. For this reason, the protocol says “number of samples + 10%” in some steps.

21. Place the tube with beads in a magnetic rack, wait until beads are collected and remove the clear supernatant.
22. Wash the beads with RIPA thrice.
  a. Add 800 μL of RIPA.
  b. Resuspend beads by inverting tubes 30 times and vortex 3–4 s at low setting. Pulse spin to eject buffer from the lid (no more than 1000 g for 5 s). Place in the magnetic rack until solution clears and remove the supernatant.
  c. Repeat steps 22a and 22b two more times.
23. Add 200 μL × (number of samples + 10%) of RIPA buffer to the tubes with beads, resuspend by inverting tube 30 times and vortex 3–4 s at low setting. Pulse spin to eject buffer from the lid (no more than 1000 g for 5 s).
24. For every sample, aliquot 200 μL of beads solution into labeled Eppendorf tubes.
25. Add 2 μg of anti-TOP1 antibody (ab109374) to each tube with beads and incubate them by gentle rotation (20 rpm) at 4°C for at least 3 h.

***Note:*** If you have prepared material for negative control, instead of the TOP1 antibody, add 2 μg of IgG antibody to the corresponding tubes.

### Assessing fragmentation on an agarose gel

**Timing: 2 h**

One of the easiest ways to check DNA fragmentation is by agarose gel electrophoresis.

***Alternatives:*** Other approaches, for examples capillary-based methods such as Agilent Bioanalyzer or TapeStation can be used as well, but they will take longer and are more expensive.

26. Prepare 0.6% agarose (Thermo Fisher, 16500500) in Tris-acetate-EDTA (TAE) buffer and cast a gel suitable for your number of samples.
27. To each 20 μL sample aliquot (from step 19) add 20 μL of TE-SDS 0.5% supplemented with 1 μL of Proteinase K (NEB, P8107S).

***Note:*** For convenience prepare a master mix with 20 μL of TE-SDS 0.5% × (number of samples + 10%) + 1 μL of Proteinase K × (number of samples + 10%) and add 20 μL of the master mix.

28. Incubate in a thermal mixer for 10 min at 60°C and 1500 rpm to release the DNA.
29. Remove the tubes from the thermal mixer. Add 4 μL of TE-NaCl (0.5 M) to neutralize the SDS, incubate at 25°C for 1 min.
30. Add 45 μL Phenol:Chloroform:Isoamyl Alcohol (Sigma P2069).

**CRITICAL:** Handle Phenol:Chloroform with extra care. Work only within a fume hood. Dispose contaminated tubes in a specialized chemical waste container.

31. Vortex the samples at the maximum setting for 5 s at 25°C.
32. Centrifuge at 6000 g for 5 min at 25°C to separate the phases.
33. Carefully collect 40 μL of the aqueous phase in a new Eppendorf tube.

***Note:*** Start by putting the tip of the pipette perpendicular to the surface of liquid and lean it while collecting the aqueous phase with the pipette tip. At some point the aqueous phase will form a bubble on the side of the tube, which is easier to collect with a pipette. If you accidentally collect the lower organic phase or interface, follow troubleshooting Problem 4: Organic phase.

34. Dilute RNase (Roche, 11119915001) 1:10 in TE, add 2 μL to each sample and incubate for 10 min at 37°C.
35. Take 18 μL of each sample and add 2 μL of DNA gel loading buffer 10×.

***Note:*** We recommend using a reduced amount of dye in the DNA gel loading buffer because during the gel run the bromophenol blue can overlap with the fragments of interest, interfering with the visualization under UV light.

36. Load 6 μL of gene ruler mix (1 μL of gene ruler (Thermo Fisher, SM0311), 1 μL of DNA gel loading buffer 10 × and 4 μL of deionized water) into the first well and 15 μL of samples + DNA gel loading buffer into the other wells.

***Note:*** Scale down the amount of loaded sample if you have lower volume of wells.

37. Run the gel at 200 V for 20 min in Bio-Rad Wide Mini-Sub Cell GT System (adjust the time and voltage for other gel chambers accordingly) or until the bromophenol blue reaches about 2/3 of the gel.

38. Stain the gel with Ethidium Bromide (EtBr) (Sigma, E1510).
  ***Note:*** Post-staining is preferable as it results in a clearer image.
  ***Alternatives:*** You can also use SYBR™ Green I Nucleic Acid Gel Stain (Thermo Fisher Scientific, S7563) instead of EtBr as a safer and more sensitive alternative for DNA staining.
  a. Transfer the gel in special EtBr container (work under the fume hood). Pour approximatively 100 mL of TAE + EtBr (1 μg/mL) in the container with the gel.
    ***Note:*** Adjust the volume depending on the size/height of the gel to cover the gel completely with TAE + EtBr.
  b. Incubate for 30 min on a rocking platform.
    **CRITICAL:** Cover the container with aluminum foil as EtBr is light sensitive and make sure that the rocking is gentle, to avoid spills of EtBr.
  c. Image the stained DNA in a Bio-Rad Gel Doc EZ Imager and check the fragment size.
    ***Alternatives:*** You can use other gel imagers.
    **CRITICAL:** Do not touch anything with the gloves you used to work with EtBr and trash them every time you are not working with EtBr.

Trash the gel in a specialized EtBr chemical disposal. Rinse with water and 70% Ethanol all the containers contaminated with EtBr.

**Figure 1:**
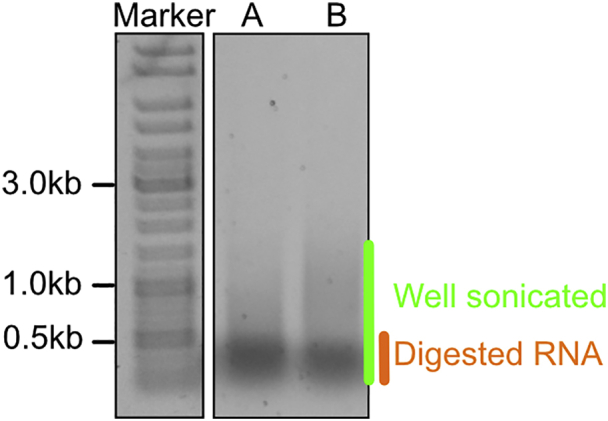
Example of fragment size distribution after sonication analyzed by gel electrophoresis In samples A and B, most of the fragments are below 1000 bp (highlighted by green). The band highlighted in orange represents digested RNA products. RNA is known to precipitate together with DNA and protein-DNA adducts during steps 12–14 (Kiianitsa and Maizels, 2013).

39. The DNA fragment size should be below 1000 bp (Figure 1). If the fragment size is bigger than 1000 bp, follow troubleshooting Problem 5: Sonication.

### Immunoprecipitation with anti-TOP1 antibody, part II

**Timing: includes 1 h hands-on time, 10 to 14 h incubation (which we suggest to perform overnight), on the next day 1 h hands-on time and 4 h incubation**

The immunoprecipitation step is similar to what it is performed in a standard ChIP protocol except that the washing steps are done in solutions with SDS 0.1%, to preserve the TOP1ccs.

40. If you haven’t already prepared the antibody-beads complexes from the section immunoprecipitation with anti-TOP1 antibody, part I: then follow this section first before continuing.
41. Prepare sample for IP.
  a. Spin the sonicated sample for 10 min, max speed at 4°C and transfer the supernatant to a fresh 1.5 mL tube.
  b. Fill up all samples to 1040 μL with TE-SDS 0.1% + protein inhibitors cocktail + AEBSF.
  c. Transfer 40 μL of sonicated sample to a new tube and add 2 μL SDS 10%. This will be used as Input (4%) to generate a standard curve for the qPCR.
  d. Take the rest (1000 μL) of sonicated sample and add 250 μL of RIPA 5× to equilibrate to RIPA buffer. Store on ice until addition to the beads.
42. Wash the antibody-beads complexes (from step 25) with 800 μL of cold phosphate buffered saline (PBS).
  a. Pulse spin tube and place it onto a magnetic rack. Once the solution clears remove supernatant with unbound antibody and add 800 μL of cold PBS.
  b. Wash the beads by inverting the tube 30 times and vortex 3–4 s at low setting. Pulse spin to eject buffer from the lid.
  c. Place tube onto the magnetic rack, wait until the solution clears and beads are collected on the side of the tube and remove supernatant.

***Note:*** The PBS wash is very important to increase the efficiency of the IP by removing the unbound antibody.

**CRITICAL:** Make sure to have the samples prepared and ready before you remove the PBS to avoid drying of the beads.

43. Add the samples (already diluted in RIPA in step 41d) to the antibody-beads complexes.
44. Incubate for 10–14 h at 4°C.

**Pause point:** We suggest performing the incubation overnight. Do not incubate longer than 16 h.

45. Wash the immunocomplexes.
  ***Note:*** This step strongly affects the yield and ratio $\left(\frac{MG132+CPT}{untreated}\right)$ of the IP. Make sure to treat all the samples consistently and to work on ice, avoiding warming up the samples.
  a. Place the tube onto the magnetic rack, remove supernatant and wash the beads with 800 μL of cold RIPA. Resuspend the beads by inverting the tube 30 times and vortex 3–4 s at low setting. Pulse spin to eject buffer from the lid. Put the tubes on the magnetic rack and discard the supernatant.
  b. Repeat step 45a but with RIPA300.
  c. Repeat step 45a but with LiCl-SDS 0.1%.
  d. Repeat step 45a but with TE-SDS 0.1%.
46. Place the tubes on the magnetic rack and remove as much buffer as possible without disturbing the beads. Add 100 μL of TE-SDS 0.5% buffer + 2 μL Proteinase K to the beads. Add 60 μL of TE-SDS 0.5% buffer + 2 μL Proteinase K to the input samples.

***Note:*** For convenience prepare a master mix with 100 μL (60 μL for input) of TE-SDS 0.5% × (number of samples +10%) + 2 μL of Proteinase K × (number of samples +10%) and add 100 μL (60 μL for input) of the master mix.

47. Incubate the beads and inputs in a thermal mixer at 60°C and 1500 rpm for at least 4 h.

### DNA purification and quantification

**Timing: 3 h**

**Figure 2:**
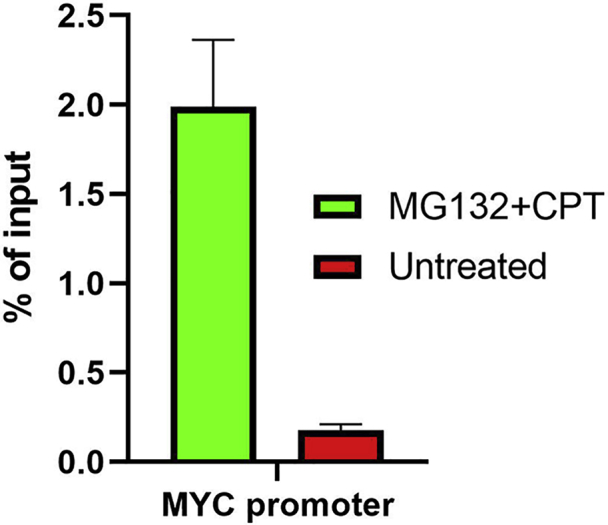
TOP1ccs enrichment determined by TOP1 CAD-qPCR in HCT116 cells represented as percentage of input (n=2, error bars denote standard deviation, SD) Primers amplify a region in the MYC promoter. The signal from IP with IgG is on average 0.001%.

Perform DNA purification with PCR purification kits and quantify DNA concentration with Qubit Fluorometer (Invitrogen, Q33216). Before going forward with library preparation, it is important to ensure a sufficient DNA yield. Based on our experience, we suggest at least 1–10 ng of DNA for every sample, and a ratio $\left(\frac{MG132+CPT}{untreated}\right)$ above 7 based on qPCR results (Figure 2).

**Pause point:** Purified DNA is very stable and can be kept at −20°C for long term storage.

***Note:*** DNA purification can be also performed by Phenol: Chloroform and Ethanol precipitation. This will add one extra day to the protocol.

48. Purify the samples with a MiniElute PCR purification kit (QIAGEN, 28006) (link).
  a. Add 500 μL of PB buffer to each 100 μL sample (directly on the beads) and input, mix well and pulse spin to eject buffer from the lid.
  b. Place on a magnetic rack, let the beads collect on the side of the tube and once the solution clears transfer 600 μL mixture of buffer and sample to the QIAquick column placed in a 2 mL collection tube.
    ***Note:*** Due to the viscosity of the buffer, the collection of beads to the side of the tube will take longer than in previous steps.
  c. Apply the sample to the column and centrifuge at 17,900 g for 60 s.
  d. Discard the flow-through and place the column back in the tube.
  e. Add 750 μL of PE buffer to the tube and wash the column by spinning at 17,900 g for 60 s.
  f. Remove the flow-through and centrifuge once more to remove any residual PE.
  g. Place the column in a 1.5 mL Eppendorf tube to collect the purified sample.
    ***Note:*** Cut off the cap from the tube before placing a column in it. If not removing the cap, make sure to transfer the eluted sample in a fresh tube to avoid contamination by the dirty lid.
  h. Add 10 μL of EB buffer to the center of the column membrane and let the membrane soak for 5 min at 25°C.
  i. Centrifuge the sample through a column at 17,900 g for 60 s to collect the purified sample at the bottom of the tube.
  j. Repeat steps 48h–i once in the same tube, to recover eventual traces of DNA from the column membrane.
  k. Transfer each purified sample to a new 1.5 mL Eppendorf or a PCR tube.
49. Quantify the DNA concentration with Qubit® dsDNA HS Assay Kit (Thermo Fisher Scientific, Q33230) (link). If the yield is too low (<1 ng) see troubleshooting problem 6: Low yield.

***Note:*** Quantification with Qubit is recommended as this step is important to calculate the volume needed for library preparation.

50. Run a qPCR using primers for the MYC promoter and calculate the ratio $\left(\frac{MG132+CPT}{untreated}\right)$ (Figure 2). If the ratio is low, see troubleshooting Problem 7: Low ratio $\left(\frac{\text{MG}132+\text{CPT}}{\text{untreated}}\right)$.

***Note:*** MYC promoter is defined as the region within 300 bp upstream of the TSS.

***Note:*** Elevated levels of TOP1ccs are known to engage with DNA upstream TSSs of highly expressed genes (Baranello et al., 2016; Kouzine et al., 2013a). Therefore, the MYC promoter was chosen as a positive control.

***Note:*** The MYC promoter should work as a positive control in all cell lines where MYC is highly expressed.

***Note:*** When designing additional primers for qPCR before library preparation, we advise to select regions upstream the TSSs of highly expressed genes.

***Note:*** The qPCR will work with conventional primers. In the first qPCR cycle the strand complementary to the TOP1cc (with free ends) will anneal with the primer and will be subjected to polymerase synthesis.

**Table:** PCR reaction master mix

Reagent	Amount
DNA template	2 μL
Sybr Green Master Mix (Thermo Fisher, 4385618)	7.5 μL
Primer FW 4 μM	1.875 μL
Primer RV 4 μM	1.875 μL
ddH_2_O	1.75 μL

**Table:** Signal and noise primers

Primer	Forward primer 5ʹ>3′	Reverse primer 5ʹ>3′
MYC promoter	GGACTCAGTCTGGGTGGAAGG	AAGGAGGAAAACGATGCCTAGA

**Table:** PCR cycling conditions

Steps	Temperature	Time	Cycles
Initial Denaturation	98°C	30 s	1
Denaturation	95°C	3 s	40 cycles
Annealing and extension	60°C	30 s	
Denaturation	95°C	15 s	1
Final extension	60°C	1 min	1
Hold	4°C	Forever

### Library preparation and sequencing

**Timing: 3 h**

We provide an example of one of the library preparation strategies, ThruPLEX DNA-seq kit, which we have used with this protocol. You can skip this section if you choose another library preparation kit. However, please read the information below marked **CRITICAL**.

**Figure 3:**
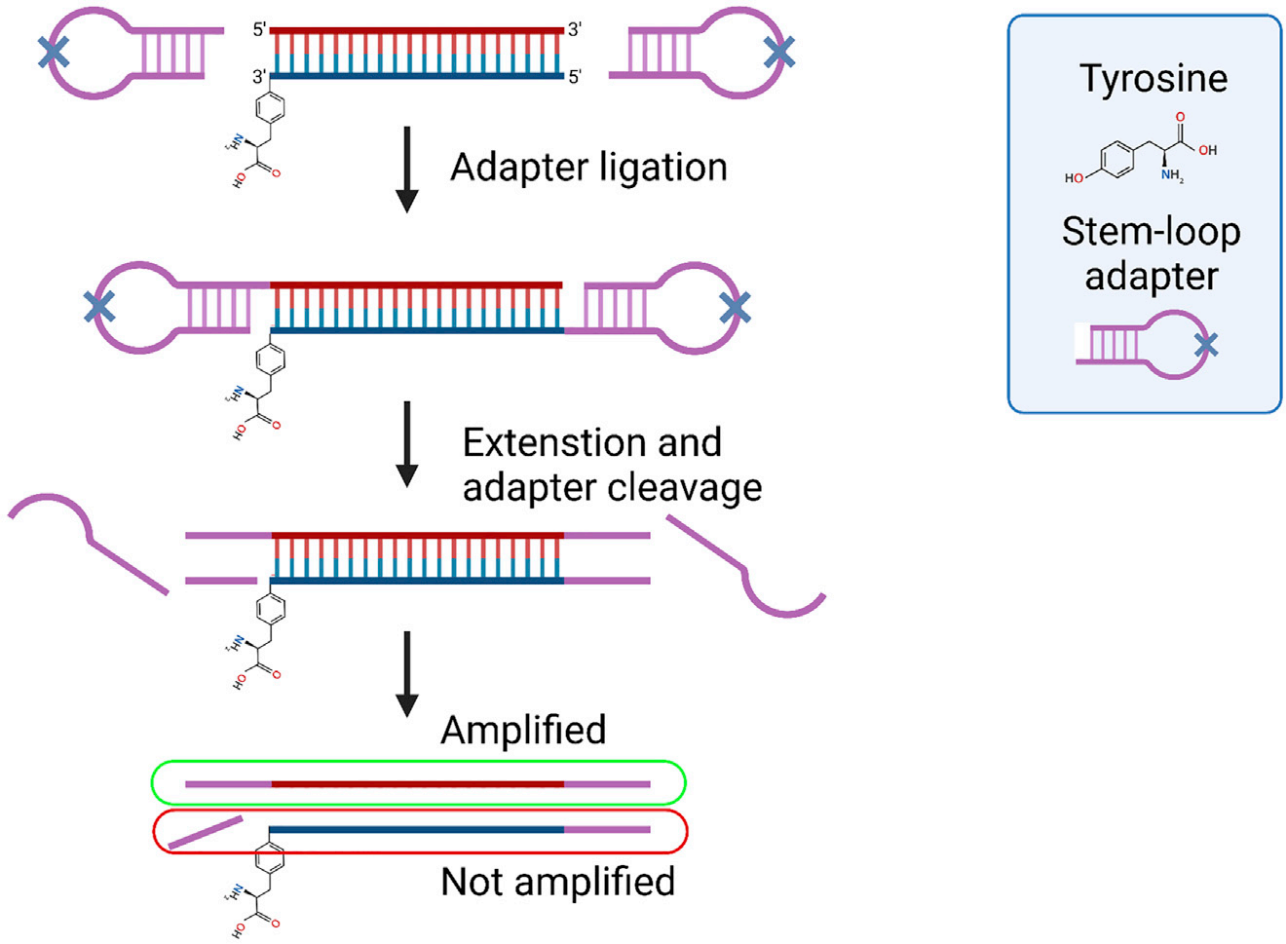
ThruPLEX library preparation works with TOP1 tyrosyl residue bound to the 3′ of a DNA molecule

**CRITICAL:** During the catalytic cycle TOP1 cleaves the phosphodiester backbone of the DNA and forms a covalent link between its tyrosyl residue and the 3′ end of the DNA molecule. This covalent link can block adapter ligation during library preparation. Thus, it is required a suitable kit for library preparation. In case of ThruPLEX DNA-seq protocol, stem-loop adapters are ligated to the 5′ end of DNA. Even with 3′ end blocked by a covalently bound tyrosyl residue, the library preparation will work for the complementary strand (Figure 3).

***Note:*** If you aim to detect TOP2ccs, after IP with anti-TOP2 antibody and DNA purification, an additional step of Exonuclease VII (NEB, M0379S) treatment and purification is required for removing the covalently bound tyrosyl residue which can block both 5′ ends of DNA and completely prevent adapter ligation and amplification during library preparation (Das et al., 2022).

51. Based on the Qubit DNA quantifications (step 49) take equal DNA amounts of each sample and transfer to 200 μL PCR tubes. The volume should not exceed 10 μL and the optimal DNA amount is 1–10 ng. Fill up all the samples to 10 μL with EB (10 mM Tris-Cl, pH 8.5).
52. Proceed with library preparation following ThruPLEX DNA-seq kit (Takara, R400676) protocol (link).

***Note:*** One TOP1cc IP with 1×10^7^ cells usually requires 10–12 cycles of PCR amplification during library preparation.

**Figure 4:**
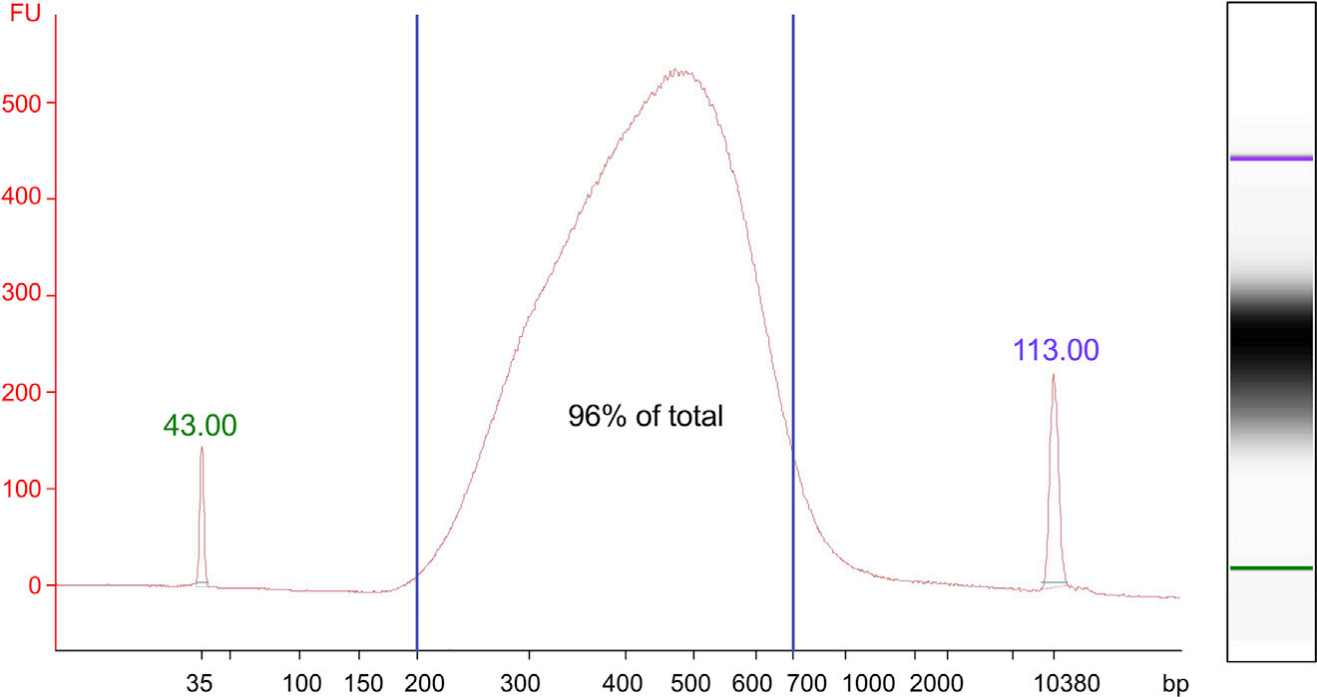
Bioanalyzer electropherogram (left) and gel (right) after library size selection 96% of the DNA is in the range 200–700 bp (blue lines).

53. Analyze the samples via capillary electrophoresis using a Bioanalyzer (Agilent, G2939BA) with the Agilent High Sensitivity DNA Kit (Agilent, 5067-4626) and check the fragment size distribution. If there is no visible DNA population, follow troubleshooting Problem 8: DNA amplification.
54. Perform library size selection using AMPure XP beads (Beckman, A63880).
  ***Note:*** Before starting, keep the beads at 25°C for at least 30 min and mix them well before pipetting.
  a. Add EB buffer to the library to obtain a final volume of 100 μL.
  b. Add 65 μL of AMPure XP beads to each sample.
    ***Note:*** Perform all pipetting of AMPure XP beads using filter tips with the tip cut off using sterile/clean scissors to prevent clogging of the tip and applying shearing forces on the beads.
  c. Mix carefully by pipetting up and down at least 10 times.
    ***Note:*** As the beads are viscous, pipette the entire volume slowly and do not vortex. Vortexing can generate bubbles and affect downstream handling.
  d. Incubate at 25°C for 8 min.
  e. Place the tube on the magnetic rack for at least 10 min or until the solution clears completely.
  f. Collect the supernatant (fragments < 700 bp) and transfer into a fresh tube prefilled with 40 μL of beads.
  g. Mix carefully by pipetting up and down at least 10 times.
  h. Incubate at 25°C for 8 min.
  i. Place the tube on the magnetic rack for at least 10 min or until the solution clears completely.
  j. Remove the supernatant containing small DNA fragments including primers and primer dimers.
  k. Add 200 μL of freshly prepared 80% Ethanol to the remaining beads, wait 30 s and remove the Ethanol.
  l. Repeat step 54k once.
  m. Spin briefly and remove any remaining drops of Ethanol.
  n. Let the pellet dry for 12 min or until tiny cracks become visible in the beads.
  o. Remove from the magnetic rack and resuspend the beads in 20 μL of EB by mixing carefully with a pipette until the solution becomes homogenous without beads clumps.
  p. Place the tube on a magnetic rack for at least 5 min or until the solution clears.
  q. Transfer the supernatant containing the library to a new tube avoiding beads carry over. This process should have selected for library fragments between 200–700 bp (Figure 4).
    ***Note:*** With some magnetic racks it is extremely difficult to avoid beads carryover, therefore reduce the volume of transferred supernatant and leave some supernatant with beads in the tube or pass through the magnetic rack 1-2 more times (step 54p-q) if the beads are still in the solution.

55. Check the efficiency of size selection via capillary electrophoresis using a Bioanalyzer and Agilent High Sensitivity DNA Kit (link).

***Note:*** If primers/dimers or longer fragments were not properly filtered, follow troubleshooting Problem 9: Primers/dimers.

56. Quantify final libraries using the Qubit dsDNA HS kit (link).
57. Make an equimolar pool of libraries based on the fragment size and DNA concentrations from Bioanalyzer and Qubit respectively. The optimal molarity of the final library pool for NextSeq550 (Illumina, SY-415-1002) is 4 nM.

***Note:*** Here we describe how to perform sequencing using NextSeq550. If you are sequencing with another machine, follow the manufacturer’s manual.

***Note:*** The final diluted libraries have low molarity, we advise to prepare the library pool right before sequencing and avoid storage for long periods. The library pool can be stored for a day at −20°C.

58. Take an aliquot of library pool for sequencing with NextSeq550.
59. Follow NextSeq550 Denature and Dilute Libraries Guide from Illumina (link).
60. At the end of a sequencing run, download BCL or fastq files for further processing.

## Expected outcomes

**Figure 5:**
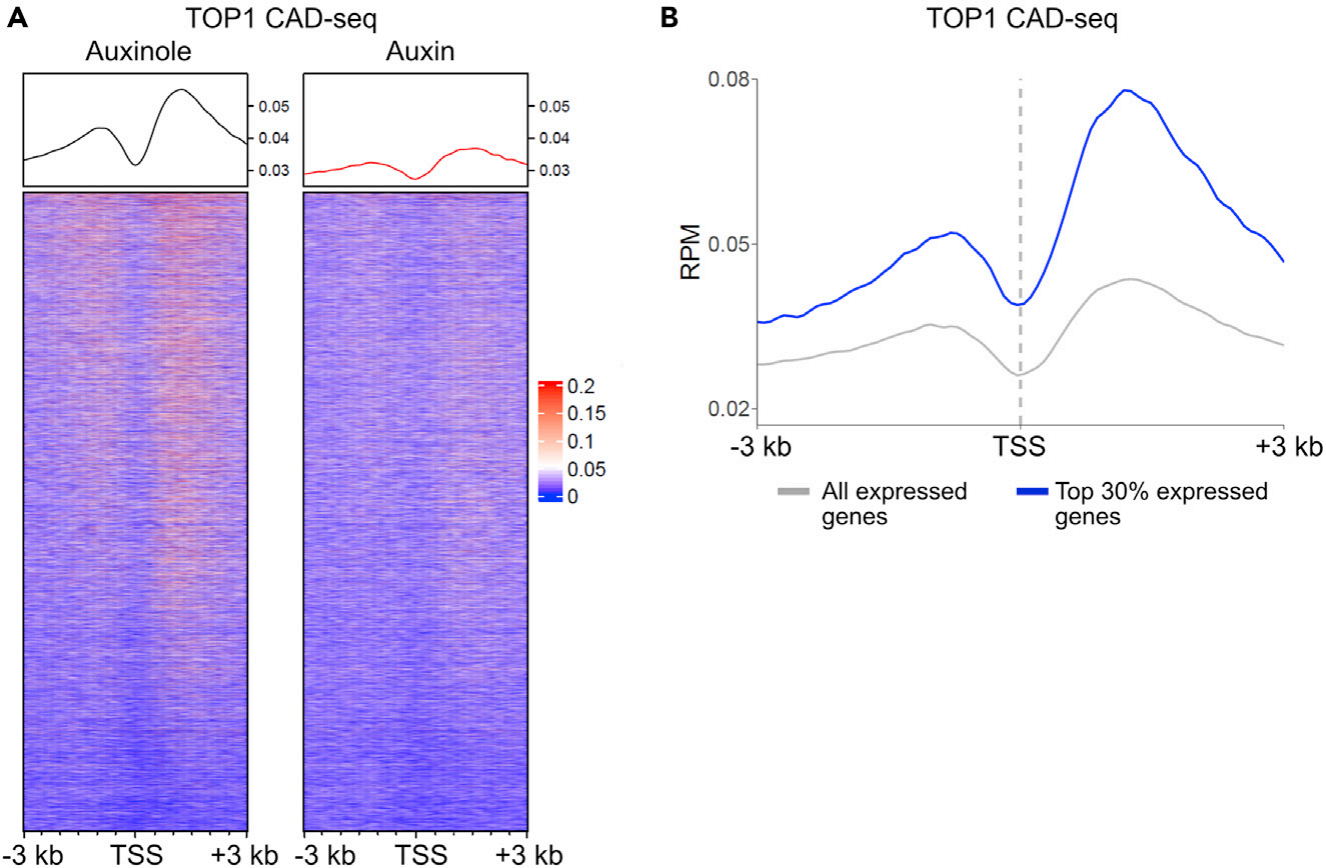
TOP1 CAD-seq signal at TSSs of genes in the presence or absence of the transcription factor MYC (A) MYC degradation reduces TOP1 activity in the region around the TSS. Heatmap (visualized with EnrichedHeatmap R/Bioconductor package (Gu et al., 2018)) of TOP1 CAD-seq signal (represented as Reads Per Millions, RPM) at TSSs of protein coding genes in K562MYC_mAID cells (Muhar et al., 2018) treated with Auxin (30 min, 500 μM) or Auxinole (100 μM), MG132 (30 min, 10 μM) and CPT (20 μM) in the last 5 min. The Auxin competitor Auxinole prevents leaky degradation of MYC. The pattern we see with TOP1 CAD-seq (left) recapitulates the results of Top1-seq published in our previous study (Baranello et al., 2016), where the TOP1ccs bound to TSSs are sparse but increase downstream of the TSSs. This is because TOP1cc formation is suppressed until after pause release when the phosphorylated-RNAPII stimulates TOP1 activity. As MYC affects the levels of TOP1ccs (Das et al., 2022), we observe a reduction in TOP1 activity around the TSSs upon MYC degradation (right). (B) Average TOP1 CAD-seq profile (RPM) at TSSs comparing all expressed genes and the top 30% expressed genes (based on RNA-seq from (Goldenson et al., 2020)). TOP1 CAD-seq signal is higher at the top 30% expressed genes indicating that TOP1 activity is proportional to the level of gene expression (Baranello et al., 2016).

TOP1 CAD-seq is a fast and reproducible high-throughput method for mapping TOP1ccs. Essentially, the type of expected data is similar to a ChIP-seq dataset, except that the location of TOP1-DNA sites (i.e., sites where the enzyme is active) is detected (Figure 5). Typical recovery from an IP is 0.1–1 ng/μL (Table 1) with a total yield of up to 10–20 ng in 15–20 μL volume. The expected number of raw reads should be at least 20 M per sample in at least 2 replicas.

***Note:*** The duplication level of raw fastq files can be high, around 40% due to possibly low recovery and PCR amplification during library preparation.

***Note:*** The overall mapping is expected to be >90%, lower mapping to the genome of interest might suggest exogenous contamination.

**Table 1:** Ranges of acceptable IP recovery, number of sequencing reads, and mapping statistics for TOP1 CAD-seq

IP recovery	Concentration before purification, ng/μL		Concentration after purification, ng/μL	
TOP1cc	0.1–1		0.1–1	
**.fastq**	**Duplicates, %**		**Read number, millions**	
TOP1cc	10–40		20–40	
**Alignment**	**0 times, %**	**1 time, %**	**>1 times, %**	**Overall, %**
TOP1cc	0–10	60–90	10–30%	>90
**.bam Deduplicated**	**Duplicates, %**		**Read number, millions**	
TOP1cc	0–5		10–30	

## Quantification and statistical analysis

### Data processing: Sequencing analysis

**Timing: It depends on the computing power of your machine/cluster. For example, processing of the .fastq files from two samples (20 M reads each) on a laptop with i7-10750H CPU and 16 GB RAM takes around 1 h. This timing includes all the processing below except for the rarely required demultiplexing and building of a reference genome which also takes around 1 h but can be done in advance for the alignment of any number of samples**

Here we provide a quick overview of sequencing data processing for this protocol. After running the pipeline you will get .bam and .bw files without duplicates, which will be used for custom downstream analysis such as PCA, average metagenome profiles, heatmaps and other. Below we provide an analysis example performed on a cluster with 20 cores and 128 gb of RAM. You can perform the analysis on personal computers, although it will likely take longer.

1. Set up your working directory ($path) for analysis and move the required files there. Move BCL into $path/BCL folder if you need to demultiplex or .fastq into $path/fastq_merged if you have untrimmed fastq files not split by lanes. For the script below to work, each sample should be in duplicates and duplicates should have the following format: “Sample name”_1.fastq.gz, “Sample_name”_2.fastq.gz.

path= #define you working directory here

mkdir -p $path/bcl2fastq/{BCL,Reports,Stats} #if you demultiplex

mkdir -p $path/{fastq_merged,FastQC_untrimmed,fastq_trimmed,FastQC_trimmed,bam/markdup/merged,bw_RPM}

threads=20 #define a variable with number of threads available for processing

***Optional:*** If your sequencing machine did not perform demultiplexing of your library, use bcl2fastq (Illumina) tool to generate .fastq files from raw base calls.

***Note:*** Demultiplexing requires an Illumina sequencing sample sheet. Place it in $path/bcl2fastq/BCL/SampleSheet.csv. To generate the sample sheet please refer to bcl2fastq guide.

bcl2fastq -R $path/bcl2fastq/BCL \

  --sample-sheet $path/bcl2fastq/BCL/SampleSheet.csv \

  --output-dir $path/fastq_merged \

  --reports-dir $path/bcl2fastq/Reports \

  --stats-dir $path/bcl2fastq/Stats \

  --no-lane-splitting \

  --barcode-mismatches 1 \

  -p$threads-w$threads-r$threads

2. Quality check your samples with FastQC and MultiQC (Ewels et al., 2016).

for i in $path/fastq_merged/∗fastq.gz

do

  fastqc $i -t$threads--extract –outdir $path/FastQC_untrimmed

done

cd $path/FastQC_untrimmed/

multiqc $path/FastQC_untrimmed/∗

3. Perform trimming of the adapters from the reads, remove low quality and short reads using cutadapt (Martin, 2011).

for file in $path/fastq_merged/∗fastq.gz

do

  f="$(basename $file)"

  h="${f%%.∗}"

  cutadapt -a AGATCGGAAGAGCACACGTCTGAACTCCAGTCA -j$threads--trim-n --nextseq-trim 25 -m 30 -o $path/fastq_trimmed/$h".trimmed.fastq.gz" $file

done

4. Check the quality of your trimmed reads with FastQC and MultiQC again. If needed adjust the cutadapt settings and repeat the trimming.

for i in $path/fastq_trimmed/∗fastq.gz

do

  fastqc $i -t$threads--extract --outdir $path/FastQC_trimmed

done

cd $path/FastQC_trimmed/

multiqc $path/FastQC_trimmed/∗

5. Download human reference genome hg38 from hgdownload.soe.ucsc.edu/goldenPath/hg38/bigZips/ and index the genome with bowtie2 (Langmead and Salzberg, 2012).

cd $path/ref/

bowtie2-build --threads$threads$path/ref/hg38.fa $path/ref/hg38

6. Align your reads to human reference genome using bowtie2. Sort, mark duplicates and index your .bam alignment files using picard and samtools (Li et al., 2009).

for i in $path/fastq_trimmed/∗fastq.gz

do

  f="$(basename $i)"

  h="${f%%.∗}"

  echo "Aligning $f ..."

  echo "Writing into file $h.sorted.bam"

  bowtie2 --threads$threads-x $path/ref/hg38 $i | samtools view -S -b | java -Xmx128g -jar $PICARD_HOME/picard.jar SortSam I=/dev/stdin O=$path/bam/$h.sorted.bam SORT_ORDER=coordinate

  java -Xmx128g -jar $PICARD_HOME/picard.jar MarkDuplicates INPUT=$path/bam/$h.sorted.bam OUTPUT=$path/bam/markdup/$h".markdup.sorted.bam" METRICS_FILE=$path/bam/markdup/$h".metrics.txt" REMOVE_DUPLICATES=true CREATE_INDEX=true

done

***Optional:*** If you want to have bam files of merged duplicates use picard MergeSamFiles.

cd $path/bam/markdup

for i in $path/bam/markdup/∗bam

do

  f="$(basename $i)"

  h="${f%%.∗}"

  p="${h%_[1-2]∗}"

  echo $p>>temp.txt

done

cat temp.txt | uniq > dup_names.txt

while read p; do

  echo "Merging "$p"_1 and "$p"_2"

  java -Xmx128g -jar $PICARD_HOME/picard.jar MergeSamFiles I=$path/bam/markdup/$p"_1.markdup.sorted.bam" I=$path/bam/markdup/$p"_2.markdup.sorted.bam" O=$path/bam/markdup/merged/$p".markdup.sorted.bam" CREATE_INDEX=true

done < dup_names.txt

7. To visualize the data, generate bigwig RPM normalized files using deeptools (Ramírez et al., 2014).

for file in $path/bam/markdup/∗bam

do

  f="$(basename $file)"

  h="${f%%.∗}"

  bamCoverage -b $file -o $path/bw_RPM/$h".RPM.bw" -bs 10 --normalizeUsing CPM -p max -v

done

8. Resulting bam and bigwig files for every sample can be further used for custom downstream analysis.

***Optional:*** Utilize deeptools or ngsplot (Shen et al., 2014) functionality to create average profiles and heatmaps for visual representation and qualitative comparisons of conditions.

***Note:***Figure 5 shows a visual representation of the processed data.

## Limitations

Although fast and specific TOP1 CAD-seq has several limitations. First, like many antibody-based methods it requires a high number of cells (∼1 × 10^7^) for a rather low yield (<10 ng), typically less than 1 ng DNA/1 million cells. In this regard, there are several factors to consider when performing the protocol. i) Although TOP1 is highly expressed in cancer cells (Heestand et al., 2017), TOP1 CAD-seq is designed to select only TOP1 molecules covalently engaged with the DNA, which represent a fraction of the total protein level. ii) Because TOP1 is a highly processive enzyme (Seol et al., 2012), the TOP1ccs are extremely transient. Treatment with CPT and MG132 allows to trap and stabilize the TOP1ccs. Harvesting in buffer containing chaotropic salts needs to be performed as quickly as possible as that might affect the total yield. Inclusion of SDS in all the buffers is also key to keep the TOP1ccs stably bound to DNA during the procedure. iii) Sonication should be performed with care. Over-sonicating the sample might cause dissociation of TOP1ccs from the DNA, thus reducing the total yield. Second, this method provides a “snapshot” of TOP1ccs on the genome from a population of cells at a specific timepoint, therefore it cannot be used to look at TOP1 dynamics in individual cells. Finally, even though the treatment with the proteasome inhibitor MG132 is performed for a short time, we cannot rule out completely that it might affect other biological processes.

## Troubleshooting

### Problem 1

Precipitation.

Sample precipitates during the pre-sonication (step 10).

### Potential solution

Make sure that when sonicating the samples, the tubes are submerged in water and that no ice is in direct contact with the tube. If the sample precipitates, place it in a rack on top of the ice until the precipitation dissolves. Make sure that the tube does not directly contact the ice. It is not recommended to warm up the sample, since that can promote degradation of the proteins.

### Problem 2

Foaming.

Sample foamed during the sonication (step 10).

### Potential solution

Stop the sonication, keep a note about how many rounds of sonication you did for the sample. Pulse spin for 30 s at 4°C. If the foam persists, put the tube in a rack on top of ice and wait until the foam dissipates, it can take up to 10 min depending on the amount of foam. Ensure that the tube is not tilted during sonication to create a small and even surface of the liquid.

### Problem 3

Resuspension.

The DNA pellet does not resuspend (step 17).

### Potential solution

Incubate the tube at 4°C with gentle rotation for additionally 30 min or until the pellet is fully dissolved. If necessary, try vortexing the sample for a few seconds.

### Problem 4

Organic phase.

Organic phase or interphase is collected during the extraction of the aqueous phase (step 33).

### Potential solution

Transfer aqueous phase with traces of other phases back in the same tube and repeat the vortex and centrifugation of the sample. As an additional precaution reduce the volume of the collected aqueous phase to avoid disturbing the other phases.

### Problem 5

Sonication.

**Figure 6:**
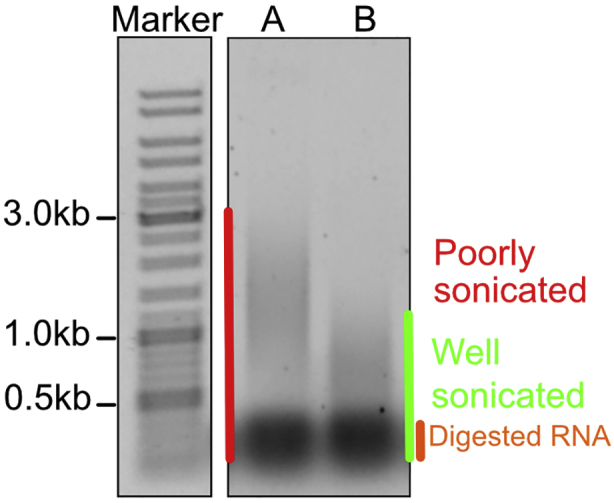
Agarose gel electrophoresis of samples after sonication (step 18) Sample A requires additional sonication while sample B is well sonicated. The band highlighted in orange represents digested RNA products. RNA is known to precipitate together with DNA and DNA-protein adducts during steps 12–14 (Kiianitsa and Maizels, 2013).

Some of the samples are not fully sonicated (step 39) (Figure 6).

### Potential solution

Repeat Sonication with Covaris: and Assessing fragmentation on an agarose gel: sections for these samples. Depending on the length of the fragments add between 1 to 5 extra min of sonication. For example, sample A in Figure 6 might require additional 3 min of sonication. As reported in (Kiianitsa and Maizels, 2013), the pellet after the precipitation step will include DNA, RNA and DNA-protein adducts. The highly pronounced low molecular weight band in Figure 6 (marked orange) might consist mostly of digested RNA products.

### Problem 6

Low yield.

The yield is lower than expected (step 49).

### Potential solution

Scale up number of cells, antibody amount, make sure not to lose the pellet during the precipitation or the washes and not to warm up the tube during the full protocol.

### Problem 7

Low ratio $\left(\frac{MG132+CPT}{untreated}\right)$.

The yield is high, however the ratio $\left(\frac{MG132+CPT}{untreated}\right)$ is significantly lower than 7 (step 50).

### Potential solution

**Figure 7:**
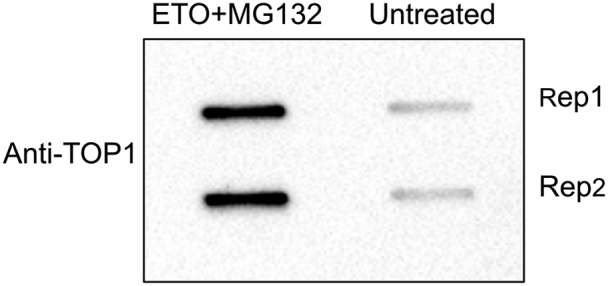
Dot blot showing TOP1ccs from HCT116 cells treated or not with CPT + MG132 1 μg DNA loaded per sample (technical replicas, n=2).

***Note:*** If you plan to perform Dot Blot, remember to include extra cells (1 × 10^6^ to 3 × 10^6^) for each sample.

• Repeat the procedure described in step 45 performing two washes with each buffer instead of one wash. You can assess the background level by performing an additional Dot Blot (Kiianitsa and Maizels, 2013) (Figure 7) comparing the recovery of TOP1ccs from treated and untreated samples (see a step-by-step protocol below).

• Follow the protocol up to step 11. Take an aliquot of 300 μL for protein analysis.

***Note:*** This amount corresponds to material isolated from approximatively 3 × 10^6^ cells. If you are working with higher or lower concentration of cells, scale the volume accordingly to have between 1 × 10^6^ and 3 × 10^6^ cells in each aliquot.

• Precipitate the material following Precipitation: section except for scaling down volumes (150 μL of 100% cold Ethanol and 300 μL of Wash Buffer). Resuspend the final pellet in 200 μL of freshly prepared 8 mM NaOH in ultrapure water (instead of TE-SDS0.1%) and incubate for 30 min.
• Sonicate the DNA for 20 s with a Bioruptor sonicator (Diagenode, UCD-200) at medium intensity setting.

***Note:*** For optimal sonication efficiency place no more than 3 Eppendorf tubes in the sample holder of the Bioruptor sonicator, equidistant from each other.

***Alternatives:*** Use other sonicators if needed. Bandelin probe sonicator or Covaris will work given that the settings are adjusted accordingly.

• Quantify DNA with Qubit dsDNA HS Assay Kit.
• Per slot: digest 1 μg DNA in 100 μL buffer (+10%) with benzonase (Sigma, E8263). Use approximately 0.05 U benzonase per ng of DNA in TBS with addition of 2 mM MgCl_2_.
• Incubate for 30 min at 37°C.

**Pause point:** Proteins can be kept in the fridge for one day.

• Transfer proteins yielded by digestion of 1 μg of DNA onto a PVDF membrane by slot blotting in TBS and immunostain for TOP1 protein.
  • Cut the membrane and 2 sheets of Whatman paper to the size of the chamber.
  • Activate the PVDF membrane in MeOH for approximatively 30 s (it will turn transparent), incubate 2 min in MilliQ water, and equilibrate 5 min in TBS.
  • Soak Whatman papers in TBS.
  • Assemble the vacuum slot blotting chamber according to the manufacture’s instruction, placing 2 soaked Whatman papers under the pre-equilibrated membrane.
  • Test the set-up by loading 100–200 μL TBS to each well and applying vacuum. If the buffer is soaked in uniformly, continue with loading the samples.
  • Load 100 μL of the sample (1 μg DNA) into slots. Fill all the empty slots with 100 μL TBS and apply vacuum until the sample is soaked in. Keep the vacuum on for a few minutes but do not let the membrane dry out.
    ***Note:*** We recommend blotting each sample twice because blotting variations can occur (Figure 7).
  • Take the membrane out of the slot blot chamber and stain proteins with ponceau red.
    ***Note:*** This step is important not only for visualization, but it also fixes proteins on the membrane.
  • Rinse off the remaining ponceau stain with MilliQ water.
  • Block the membrane for 30–60 min in 5% milk in TBS + Tween 0.1% (TBST).
  • Incubate with anti-TOP1 antibody (Abcam, ab109374, 1:13,000 in TBST) for 1 h.
  • Wash 4 times with TBST (5 min per wash).
  • Incubate with anti-rabbit (Abcam, ab205718, 1:35,000 in TBST+2%BSA) for at least 45 min.
  • Wash 4 times with TBST (5 min per wash).

***Note:*** Keep the incubation and washing steps short and avoid long (overnight) incubations, because proteins can be washed off the membrane.

• Detect signals using SuperSignalTM West Dura Extended Duration Substrate (Thermo Fisher Scientific, 34075) and image using a Chemidoc MP (Bio-Rad).

### Problem 8

DNA amplification.

**Figure 8:**
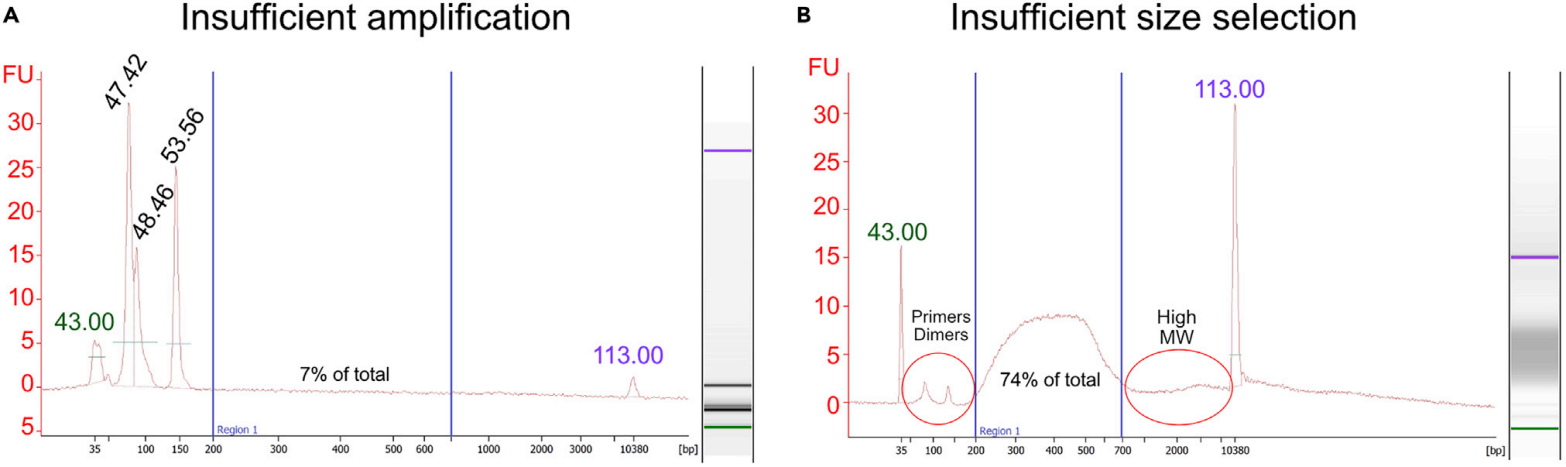
Representative bioanalyzer electropherograms (left) and corresponding gels (right) (A) Low amount of DNA in the region of 200-700 bp (blue lines). (B) Primer dimers and high molecular weight DNA (red circles) after size selection.

DNA is not visible, or signal is very low in the region 200–700 bp analyzed by capillary electrophoresis with Bioanalyzer (step 53) (Figure 8A).

### Potential solution

Proceed with additional amplification for 2–3 cycles following ThruPLEX DNA-seq kit protocol (link).

### Problem 9

Primers/dimers.

Bioanalyzer electropherogram shows residual primers/dimers or high molecular weight distribution (step 55) (Figure 8B).

### Potential solution

Repeat the selection protocol and re-check size selection (steps 54 and 55).

## Resource availability

### Lead contact

Further information and requests for resources and reagents should be directed to and will be fulfilled by the lead contact Laura Baranello (laura.baranello@ki.se).

### Materials availability

The material generated in this study is available upon request from the lead contact.

## Data Availability

All the sequencing datasets generated through this study are reported in (Das et al., 2022). Any additional information required to reanalyze the data is available from the lead contact upon request.
